# Effects of the Informed Health Choices secondary school intervention on the ability of lower secondary students in Kenya to think critically about health choices: 1-year follow-up of a cluster-randomized trial

**DOI:** 10.1186/s13063-025-08810-0

**Published:** 2025-04-07

**Authors:** Faith Chesire, Margaret Kaseje, Violet Gisore, Michael Mugisha, Ronald Ssenyonga, Matt Oxman, Allen Nsangi, Daniel Semakula, Christopher James Rose, Laetitia Nyirazinyoye, Simon Lewin, Nelson K. Sewankambo, Sarah Rosenbaum, Jenny Moberg, Andrew D. Oxman

**Affiliations:** 1 Tropical Institute of Community Health and Development, P.O. Box 4074 – 40103, Kondele, Kisumu, Kenya; 2 Institute of Health and Society, Faculty of Medicine, University of Oslo, Oslo, Norway; 3 School of Public Health, College of Medicine and Health Sciences, University of Rwanda, Kigali, Rwanda; 4 Makerere University, College of Health Sciences, Kampala, Uganda; 5 Centre for Epidemic Interventions Research, (CEIR), Norwegian Institute of Public Health, Oslo, Norway; 6 Department of Health Sciences åLesund, Norwegian University of Science and Technology (NTNU), Ålesund, Norway; 7 Health Systems Research Unit, South African Medical Research Council, Cape Town, South Africa

**Keywords:** Critical thinking, Critical health literacy, Secondary school curriculum, Treatment claims, Health information, Kenya

## Abstract

**Introduction:**

The Informed Health Choices (IHC) secondary school intervention aimed to teach students to assess claims about treatments. This follow-up of a cluster randomized trial assessed the retention of knowledge and the application of the nine prioritized IHC key concepts 1 year after the intervention.

**Methods:**

We conducted a random assignment of 80 secondary schools in Western Kenya into either the intervention (*n* = 40) or control (*n* = 40) group. Both groups adhered to the standard curriculum. Teachers from the intervention group were invited to participate in a 2-day training workshop and were granted access to “Be Smart About Your Health” digital resources, comprising 10 lessons. These lessons, focused on nine prioritized IHC concepts, delivered over a single school term from May to August 2022. The digital resources were accessible online via smartphones or computers and could also be downloaded for offline use. The primary outcome measure, assessed at the end of the school term and again after 1 year, was the percentage of students achieving a passing score (defined as ≥ 9 out of 18 correct answers) on the “Critical Thinking about Health” test.

**Results:**

Out of the total 3360 students involved in the trial, 2446 (72.8%) completed the test after 1 year. Within the intervention group, 728 out of 1369 students (53.2%) achieved a passing score after 1 year, compared to 61.7% immediately post-intervention. In contrast, in the control group, 347 out of 1077 students (32.2%) had a passing score after 1 year. The adjusted difference in passing rates between the intervention and control groups after 1 year was 20.8% (with a 95% confidence interval of 13.6 to 28.0%), compared to 27.3% (with a 95% confidence interval of 19.6 to 34.9%) immediately after the intervention.

**Conclusion:**

This study demonstrates that students were able to retain knowledge and the ability to apply the IHC key concepts, 1 year after the intervention. But fewer students in the intervention group had a passing score after 1 year compared to just after the intervention. Highlighting follow-up training is likely necessary to reinforce these skills over time.

**Trial registration:**

Pan African Clinical Trial Registry, trial identifier: PACTR202204883917313. Registered on 05/04/2022.

**Supplementary Information:**

The online version contains supplementary material available at 10.1186/s13063-025-08810-0.

## Introduction

Social and mass media are rampant with claims about the benefits and harms of treatments or other health actions [1, 2]. Unfortunately, many people lack critical evaluation skills that they can apply to such claims [3–6]. Acting on unreliable health claims or ignoring reliable ones can lead to unnecessary suffering or resource wastage [7, 8], a significant concern in low-income countries. Thus, people need to learn critical thinking skills to assess health information accurately [9, 10], particularly youth who may benefit most from learning these skills for future decision-making.

Together with teachers, curriculum developers and students, we identified and prioritized nine out of the 49 Informed Health Choices (IHC) key concepts (Table 1) relevant to secondary school students in Kenya, Rwanda, and Uganda [11]. The IHC concepts are principles that people need to understand and apply when assessing claims about treatments [12]. Using an iterative human-centered approach [13], we designed and piloted, “Be Smart About Your Health” educational resources for teaching the nine IHC key concepts [14]. We developed and validated the Critical Thinking about Health (CTH) test [15] as a set of measure to assess ability of lower secondary school students to apply those concepts. The intervention consisted of two main components: 10 lessons taught using the *Be Smart About Your Health* digital resources and a 2-day training workshop for the teachers.

**Table 1:** Prioritized IHC key concepts in the secondary school resources

1. Treatments can cause harms as well as benefits
2. Large, dramatic effects are rare
3. Personal experiences or anecdotes alone are an unreliable basis for most claims
4. Treatments that are new or technologically impressive may not be better than available alternatives
5. Widely used treatments or those that have been used for decades are not necessarily beneficial or safe
6. Identifying the effects of treatments depends on making comparisons
7. Small studies may be misleading
8. Comparison groups should be as similar as possible
9. Weigh the benefits and savings against the harms and costs of acting or not

In a cluster-randomized trial, we evaluated effects of the intervention at the end of the school term in which the intervention was implemented and again a year later. We did not intervene in the control schools. At the end of the term, the intervention had a large effect on test scores 1149/1863 (61.7%) of students achieved a passing score in intervention schools compared to 511/1499 (34.1%) in the control schools (odds ratio 3.6 (95% CI 2.5–5.2), *p* < 0.0001) [16]). In this paper, we examine the outcomes evaluated 1 year after the intervention. Our aim is to assess retention of the ability to apply the IHC key concepts. Details regarding the study methods can be found in the trial protocol [17] and evaluation report [16] We conducted a process evaluation alongside the trial [18] Parallel trials [19, 20] and process evaluations were conducted in Rwanda and Uganda [21, 22].

## Methods

### Participants

**Fig. 1:**
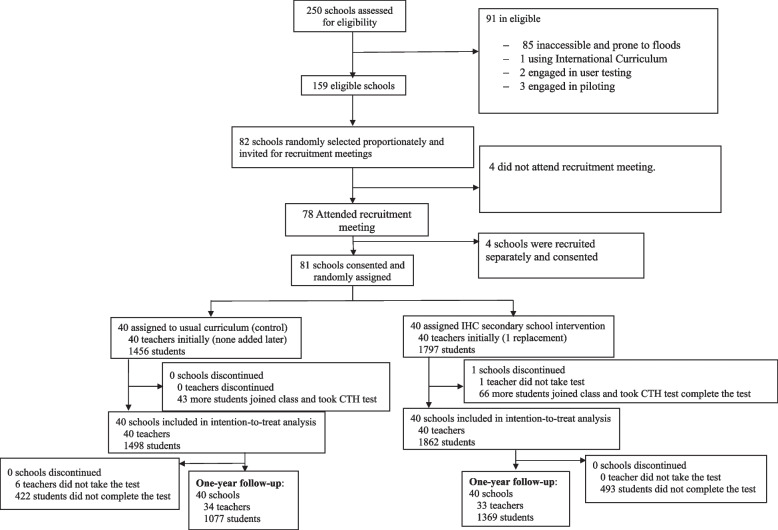
Informed Health Choices trial profile with the 1-year follow-up

Between May 11, 2022, and July 8, 2022, we recruited a representative sample of 80 secondary schools in Western Kenya (Fig. 1). To do this, we stratified the schools by ownership (private/public) and geographical location (rural/urban) and randomly selected eligible schools from each sub-county and across the four strata. These schools had characteristics like those found nationwide; the average class size in Kenyan schools ranges from 40 to 70 students, with this study showing similar averages: 46 students in control schools and 52 in intervention schools, the national age range for students is 13 to 15 years, which aligns with the age of the students who participated in the study.

In collaboration with the sub-county education directors, we introduced principals of the selected schools to the study objectives and methods and secured written consent for their schools and students to participate. The principals selected a teacher from their staff who was already teaching a stream of first-year class and who was willing to participate in the study prior to random allocation. The principals together with the selected teachers selected a first-year class to participate in the trial. We met with the teachers to acquaint them with the study objectives and sought their written consent.

### Random allocation and blinding

We randomly assigned the 80 schools to either the intervention or control group using block randomization based on ownership (public/private) and geographical location (rural/urban). An independent statistician, not involved in school recruitment, utilized *Sealed Envelope* [23] to create the allocation sequence. The principal investigator (FC) assigned unique codes to the schools and shared them with the statistician to ensure allocation concealment. The statistician then generated a randomization list, pairing unique codes with corresponding allocation groups for each school. The list remained unchanged post-randomization. Due to the intervention’s nature, blinding the research team, teachers, and students was not feasible. We informed teachers and students about the purpose of the CTH test. They were not shown the test until the end of the school term, when they completed it.

## Intervention

The intervention included providing a 2-day training workshop for teachers and digital resources to teach 10 lessons. The resources were accessible online with a smartphone or computer and could be downloaded for use offline. The lessons were designed to be taught within a single school term, taking 40 min per lesson. Standard examination time (approximately 3 h) was allocated for completing the CTH test at the end of the term and again after 1 year. For each lesson, the resources included an overview, detailed lesson plan, and background information for teachers. There were two versions of each lesson plan, one for classrooms with a projector and one for classrooms with only a blackboard and/or flipchart. The digital resources also included a teacher’s guide, glossary, training materials used in the workshop, and a set of optional printable files for teachers and students.

Five teachers who piloted the resources facilitated the teacher training workshop. The training aimed to enhance intervention teachers’ understanding of the nine key concepts and how to effectively utilize the learning resources. We invited the 40 teachers from the control schools to an introductory meeting where we outlined the study procedures, including the format of the CTH test. In the process evaluation [24], we conducted observations of the first lesson across all 40 schools assigned to the intervention arm. From these, we selected 10 schools for more intensive monitoring, where research assistants observed each of the remaining nine lessons using a standardized observation checklist. Additionally, teachers submitted a class delivery report for every lesson, using a standardized checklist.

During the study, only one teacher was replaced due to a transfer initiated by the Ministry of Education after the intervention had begun. The replacement teacher received the same comprehensive training as the original cohort of teachers. As part of the training, we emphasized the importance of confidentiality, and all intervention teachers formally agreed not to share links to or materials from the intervention with schools in the control group. We assured them of receiving the *Be Smart About Your Health* resources upon completion of the study but did not introduce the resources to them. We did not otherwise intervene in the control schools. The intervention is described using the GREET checklist in Appendix 1. [25]

### Outcomes

The CTH test (Appendix 2) [15] was administered at the end of the school term when the lessons were taught and again 1 year later to measure the students’ ability to apply the nine key concepts addressed by the intervention. The test included 18 multiple-choice questions (MCQs) with two questions addressing each of the nine key concepts. The primary outcome was the proportion of students with a passing score on the test, defined as answering at least nine out of the 18 MCQs correctly. We determined the cut-off for passing and mastery scores [26] using a combination of Nedelsky’s [27] and Angoff’s [28] methods.

Secondary outcomes included the mean difference in scores, the proportion of students achieving a mastery score (at least 14 out of the 18 MCQs answered correctly), the proportion of students answering both questions correctly for each key concept, and differences in students’ intended behaviors and self-efficacy (Appendix 2). Additional secondary outcomes included the proportions of teachers achieving passing and mastery scores, and the mean difference in their scores.

### Statistical analysis

All analyses were pre-specified, except as noted below. We estimated the sample size using the University of Aberdeen Health Services Research Unit’s Cluster Sample Size Calculator [29] as described in our previous trial report.

We estimated odds ratios and differences in means for binomial and continuous outcomes, respectively. We estimated odds ratios using mixed effects logistic regression. We estimated differences in means using mixed effects linear regression. For outcomes measured at the student level, we accounted for the cluster-randomized design using random intercepts at the level of school (the unit of randomization), and report model-based intraclass correlation coefficients (ICCs). Because there is a one–one relationship between teachers and schools, no such adjustment is necessary for outcomes measured on teachers. Except where noted below, all analyses were adjusted for the variables used in the stratified randomization (public versus private schools, and urban versus rural schools). Missing test answers were counted as wrong answers. All analyses were performed according to the intention-to-treat principle. Statistical analyses were performed using Stata 18 (StataCorp LLC, College Station, Texas, USA).

We conducted two prespecified sensitivity analyses to explore the risk of bias due to attrition. First, we conducted an inverse probability weighted (IPW) analysis where each participant was assigned a weight based on their probability of remaining in the study [30]. Weights were estimated using elastic net logistic regression where covariates could be selected from among all baseline characteristics measured on the students or teachers, as appropriate.

Second, we calculated upper and lower bounds for the mean difference in test scores using the Lee bounds approach [31]. An interval estimate whose bounds correspond to the most extreme point estimates possible under missing not at random (MNAR) attrition scenarios that maximally favor and disfavor the intervention [32]. To account for the cluster-randomized design for the students, we computed confidence intervals using imputed design effects to inflate the variances of the estimators. A design effect for a particular outcome was imputed as the ratio of the variance of the IPW effect estimate (which does account for the cluster design) to the variance of an estimate from the same model without the random intercepts term. It was not possible to adjust this analysis for the stratification variables.

To aid interpretation, we re-expressed odds ratios as estimates of risk difference, accounting for uncertainty on control odds as well as the odds ratios. We converted odds ratios from logistic regression analyses to adjusted differences using the intervention group percentage as the reference. We report 95% confidence intervals and two-sided *p*-values, where appropriate, and *p*-values are reported as inequalities for *p* < 0.0001, throughout.

Retention of what was learned by students and teachers in the intervention schools is reported as the test scores in the intervention schools after 1 year relative to the initial test scores just after the intervention. We adjusted these ratios for chance by subtracting the probability of answering questions correctly by chance alone (guessing) from each proportion for passing and mastery scores (10.76% and 0.0145% respectively), from each mean for the mean scores (33.33%), and from the proportion of students answering both questions correctly for each concept (11.11%).

To put the effect of the intervention in the context of the effect sizes reported for other interventions to improve critical thinking or learning in schools, we estimated Hedges’ g (a standardized mean difference) for the adjusted difference in students’ mean scores. This was estimated as the ratio of the adjusted difference to within-cluster standard deviation [33].

We estimated odds ratios comparing students’ ability to correctly answer both multiple-choice questions for each of the nine concepts and present these results as a forest plot. For questions about intended behaviors and self-efficacy, we report numbers and percentages of students for each response option and estimates of odds ratios comparing dichotomized responses (e.g., very unlikely or unlikely, versus very likely or likely). We re-expressed the odds ratios as risk differences as for other outcomes.

Finally, we assessed the extent to which students liked the lessons, found them easy, and found them helpful. We report numbers and percentages of students for each response option as well as for dichotomized responses (e.g., liked the lessons a little or very much versus disliked the lessons a little or a lot).

## Results

The 80 schools enrolled in the trial were retained for the 1-year follow-up assessment, providing data for the primary outcome measures and consequently included in the primary analyses. An equivalent percentage of schools in both the intervention and control groups were situated in rural areas (60%), with most of these schools being publicly owned (90%). For the 1-year follow-up, 1077 (71.9%) of the 1498 students in the control schools completed the CTH test, and 1369 (73.5%) of the 1862 students in the intervention schools completed the test (Table 2). Thirty-four (85.0%) of the 40 teachers in the control schools and 33 (82.5%) of the 40 teachers in the intervention schools completed the test.

**Table 2:** Characteristics of the participants at 1-year follow-up

		**Control schools (*N* = 40)**	**Intervention schools (*N* = 40)**
**Number of schools per district**			
	Kisumu Central	5 (12.5%)	3 (7.5%)
	Kisumu East	5 (12.5%)	5 (12.5%)
	Kisumu West	13 (32.5%)	7 (17.5%)
	Nyakach	11 (27.5%)	14 (35.0%)
	Seme	7 (17.5%)	11 (27.5%)
**Location**	Rural	24 (60.0%)	24 (60.0%)
	Urban	10 (25.0%)	9 (22.5%)
**Ownership**	Public	36 (90.0%)	36 (90.0%)
	Private	4 (10.0%)	4 (10.0%)
**Teachers**		40	40
**Completed CTH test**		34 (85.0%)	33 (82.5%)
**Education**^**a**^	Diploma	2 (5.0%)	0 (0.0%)
	Degree	30 (75.0%)	32 (80.0%)
	Masters	2 (5.0%)	1 (2.5%)
**Gender**^**a**^	Male	19 (47.5%)	20 (50.0%)
**Students randomized**		1498	1862
**Completed CTH test**		1077 (71.9%)	1369 (73.5%)
**Completed CTH test per class**		35 (29 to 41)	41 (32 to 47)
**Class size**		46 (38 to 55)	52 (45 to 62)
**Gender**^**a**^	Missing	1 (0.1%)	1 (0.1%)
	Female	644 (59.8%)	637 (46.5%)
	Male	432 (40.1%)	731 (53.4%)
**Age**^**a**^		15.5 (1.0)	15.4 (1.1)
**Performance on end of term exam**	Missing	43 (4.0%)	66 (4.8%)
	Low	695 (64.5%)	798 (58.3%)
	Moderate	603 (56.0%)	719 (52.5%)
	High	157 (14.6%)	279 (20.4%)

^a^Data are for participants who took the test

Although the proportion of students lost to follow-up was similar in both trial arms, more boys than girls were lost to follow-up in the intervention arm, and those lost scored approximately 5% lower on the initial CTH test compared to those who were not lost. Outcome data were entirely missing for 27% of the randomized students due to loss to follow-up. For teachers, outcome data were missing for 13 (16%), but the corresponding clusters of students were retained. Across trial arms, there was no clear evidence of significant differences in the distributions of baseline variables or original test scores between teachers lost to follow-up and those retained.

### Primary outcome

In the intervention schools, 728 (53.2%) of 1369 students attained a passing score (≥ 9/18 on the CTH test), compared to 347 (32.2%) of 1077 students in the control group (Table 3). The adjusted difference was 21.2% (95% CI 14.1 to 28.3%) in favor of the intervention. The proportion of students in intervention schools with a passing score after 1 year (53.2%) was lower than just after the intervention (61.7%). This corresponds to 88.3% retention, corrected for chance. Of the baseline variables, student attrition was statistically significantly associated with school ownership, student performance, and student gender. Attrition odds were lower for students attending public schools (OR = 0.46; 95% CI 0.28 to 0.76); for students with high (OR = 0.25; 95% CI 0.16 to 0.40) compared to low performance (OR = 0.52; 95% CI 0.35 to 0.75) on the initial CTH test; and for female students (OR = 0.77; 95% CI 0.61 to 0.98). Student attrition was not significantly associated with treatment assignment (*p* = 0.67). Teacher attrition was not statistically significantly associated with any of the baseline variables or treatment assignment. The ICC of 0.17 indicate moderate clustering effects, particularly for mastery scores among students due to differences between schools (clustering effect), while the remaining 83% is due to differences among students within the same school. This suggests a moderate clustering effect, meaning that the proportion of students in the control schools with a passing score after 1 year (32.2%) was only slightly lower than just after the intervention (34.1%), reflecting little change in the proportion of students with a basic understanding of the key concepts and how to apply them [24] (92.0% “retention,” corrected for chance).

**Table 3:** Main results

	**Control schools**	**Intervention schools**	**Difference (95% CI)**	**Odds ratio (95% CI)**	***p***	**ICC**
	40 schools	40 schools				
**Students**	1077	1369				
**Primary outcome**^**a**^						
Passing score (≥ 9/18)^b^	347 (32.2%)	728 (53.2%)	21.2% (14.1 to 28.3%)	2.6 (1.9 to 3.7)	< 0.0001	0.10
**Secondary outcomes**^**a**^						
Mastery score (≥ 14/18)^b^	19 (1.8%)	170 (12.4%)	10.2% (6.9 to 13.5%)	7.9 (4.2 to 15.1)	< 0.0001	0.17
Mean score^c^	40.4% (15.8)	49.6% (19.3)	9.1% (6.1 to 12.2%)		< 0.0001	0.11
**Teachers**^**d**^	34	33				
Passing score (≥ 9/18)^b^	24 (80.0%)	31 (96.9%)	16.8% (1.4 to 32.2%)	7.8 (0.9 to 69.2)	.0663	
Mastery score (≥ 14/18)^b^	7 (20.6%)	30 (90.9%)	71.9% (55.9 to 87.8%)	51.9 (10.9 to 246.7)	< 0.0001	
Mean score^c^	64.5% (17.8)	85.0% (11.7)	21.5% (14.6 to 28.5%)		< 0.0001	

Note: Data are % (SD), % (95% CI), or *n* (%). *ICC* intraclass correlation coefficient. Inverse probability weights were estimated using elastic net logistic regression

^a^The cluster design was accounted for using random intercepts at the level of school

^b^Logistic regression was used to estimate an adjusted odds ratio, which is re-expressed as an adjusted risk difference

^c^Teachers were treated as equivalent to the units of randomization (schools), so these models did not include random intercepts. The stratification variables were modelled as fixed effects in all analyses. Wald-type confidence intervals and two-sided normal *p*-values were computed in all analyses

### Secondary outcomes for students

#### The proportion of students with a score indicating mastery of the concepts

In the intervention schools, 170 (12.4%) of the 1369 students achieved a score indicating mastery of the key concepts (≥ 14/18), compared to 19 (1.8%) of the 1077 students in the control schools (Table 3). This corresponds to an adjusted mean difference of 10.2% (95% CI 6.9 to 13.5%) after 1 year, compared to 14.7% (95% CI 11.0–18.4%) just after the intervention. The adjusted difference with IPW (Table 4) was slightly less (9.9%; 95% CI 6.4 to 13.4%) than without IPW, whereas the odds ratio was slightly larger, 8.3 (95% CI 4.5 to 15.3) versus 7.9 (95% CI 4.2 to 15.1).

**Table 4:** Sensitivity analyses for main results

		**Adjusted difference (95% CI)**	**Odds ratio (95% CI)**	***p***	**ICC**
Students		2446			
**Primary outcome**^**a**^					
Students with a passing score (≥ 9/18)^b^	IPW^c^	20.8% (13.6 to 28.0%)	2.6 (1.9 to 3.7)	< 0.0001	0.12
**Secondary outcomes**^**a**^					
Students with a mastery score (≥ 14/18)^b^	IPW^c^	9.9% (6.4 to 13.4%)	8.3 (4.5 to 15.3)	< 0.0001	0.20
Mean score for students^d^	IPW^c^	8.9% (5.9 to 12.0%)	**Effect size**^f^	< 0.0001	0.13
Lee Bounds^e^		8.3 to 10.0% (3.5 to 14.4%)	0.55 (0.36 to 0.73)		
**Teachers**^g^		67			
Mean score^d^		21.5% (14.6–28.5)	**Effect size**^f^		
Lee bounds^e^		19.0 to 21.4% (8.2 to 30.5%)	1.51 (0.96 to 2.06)		

Note: Data are % (SD), % (95% CI), or *n* (%). *ICC* intraclass correlation coefficient, *IPW* inverse probability weighting

^a^The cluster design was accounted for using random intercepts at the level of school

^b^Logistic regression was used to estimate an adjusted odds ratio, which is re-expressed as an adjusted risk difference

^c^Students in each school were given a weight equal to the inverse of the proportion of students in the school that completed the CTH test

^d^Linear regression was used to estimate an adjusted difference in means

^e^These were constructed by trimming the group with less attrition at the upper and lower tails of the outcome (test score) distribution respectively

^f^Adjusted Hedges’ g

^g^Teachers were treated as equivalent to the units of randomization (schools), so these models did not include random intercepts. The stratification variables were modelled as fixed effects in all analyses. Wald-type confidence intervals and two-sided normal *p*-values were computed in all analyses

The proportion of students in intervention schools with a mastery score after 1 year (12.4%) was lower than just after the intervention (18.3%), indicating 65.1% retention, corrected for chance.

#### The mean score for students

The mean score in intervention schools was 49.6% compared to 40.4% in control schools (Table 3). The adjusted difference was 9.1% (95% CI, 6.1% to 12.2%) compared to 14.1% (95% CI 10.8 − 17.3%) just after the intervention. The adjusted difference with IPW (8.9%; 95% CI 5.9 to 12.0%) was slightly lower than without IPW. Lee bounds for the mean difference in test scores resulted in lower (worst case) and upper (best case) mean differences of 8.3 and 10.0%, respectively (95% CI, 3.5 to 14.4%). This indicates that even with the worst-case scenario, the average test score in the intervention schools was still 8.3% higher than in the control schools. The adjusted Hedges’ g was 0.55 (0.36 to 0.73).

The mean score in intervention schools (49.6%) was lower than just after the intervention (55.0%), indicating 75.1% retention, corrected for chance.

#### Results for each concept

**Fig. 2:**
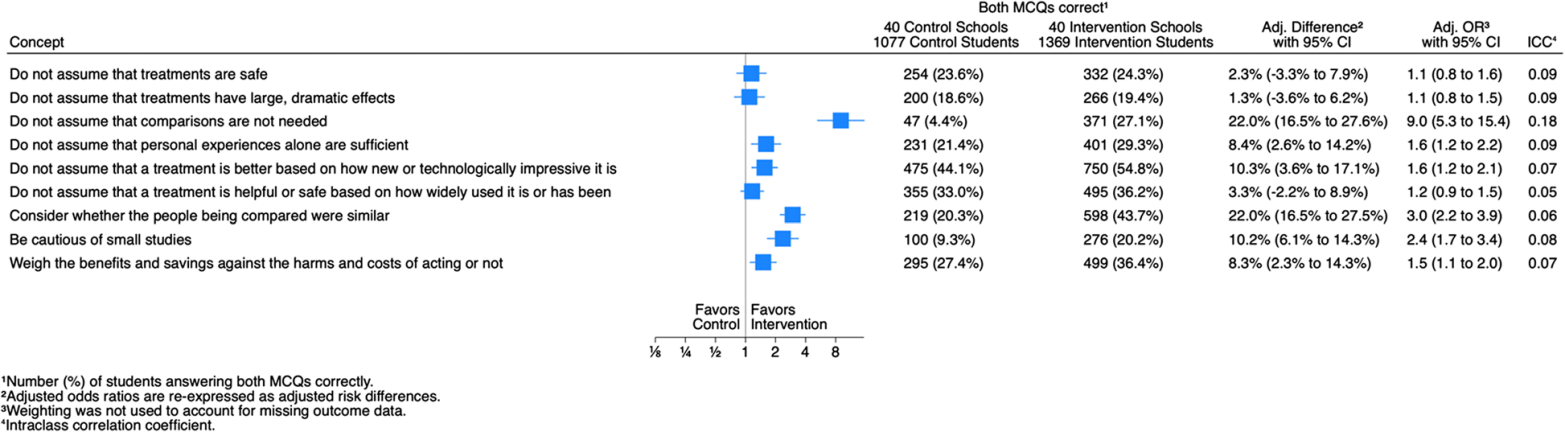
Results for each concept

The proportion of students answering both MCQs correctly for each of the nine Key Concepts was consistently higher among those in the intervention schools compared to those in control schools (Fig. 2). Adjusted differences ranged from 1.3% for the concept “Do not assume that treatments have large, dramatic effects” to 22.0% for the concept “Do not assume that comparisons are not needed.”

### Results for teachers

After 1 year, 96.9% of teachers in the intervention group achieved a passing score, compared to 80% in the control group. The adjusted difference of 16.8% (95% CI 1.4 to 32.2%) was more than just after the intervention, when the adjusted difference was 11.7% (95% CI − 2.6 to 26.0%). Just after the intervention, 97.5% of the teachers in intervention schools had a passing score, indicating retention of 95.9%, adjusted for chance.

In the intervention group, 90.9% of the teachers achieved a score indicating mastery of the key concepts (≥ 14/18) compared to 20.6% in the control group. The adjusted difference was 71.9% (95% CI 55.9 to 87.8%). This also was more than just after the intervention when the adjusted difference was 64.8% (48.3 − 81.3%). The mean score for intervention teachers was 85.0% compared to 64.5% for the control group. The adjusted difference was 21.5% (95% CI 14.6 to 28.5%) compared to 23.4% (17.9–28.9%) just after the intervention.

Lee bounds for the mean difference in test scores resulted in lower (worst case) and upper (best case) mean differences of 19.0 and 21.4%, respectively (95% CI 8.2 to 30.5%). This indicates that even with the worst-case scenario, the average teacher test score in the intervention schools was still 19% higher than in the control schools. The adjusted Hedges’ g was 1.51 (0.96 to 2.06).

### Students’ self-reported behaviors and self-efficacy

Students in intervention schools were more likely to say they would find out what a treatment claim was based on (adjusted difference 9.5%; 95% CI 4.6 to 14.5%) and if a claim was based on research (adjusted difference 13.4%; 95% CI 7.7 to 19.1%), compared to students in control schools (Table S1). These differences were slightly larger than just after the intervention when the adjusted differences were 5.4% (95% CI 1.3 to 9.5) and 11.7% (95% CI 8.3 to 15.1) respectively. There was little difference between students in intervention and control schools in the self-reported likelihood that they would agree to participate in research comparing treatments, both just after the intervention and 1 year later.

Students in intervention schools were more likely to say they find it easy or very easy to know if a treatment claim is based on research (adjusted difference 19.1%; 95% CI 13.0 to 25.3%), to find research-based information about treatments (adjusted difference 7.9%; 95% CI 2.8 to 13.0%), to judge the trustworthiness of research results (adjusted difference 11.5%; 95% CI 6.1 to 16.9%), and to know if research results are relevant to them (adjusted difference 10.1%; 95% CI 4.6 to 15.5%) (Table S2). The first two results are corresponding with the results just after the intervention. The likelihood that they would say they find it easy or very easy to judge the trustworthiness of research results and to know if research results are relevant to them was larger than just after the intervention when the adjusted differences were 4.2% (95% CI 0.4 to 8.0) and 6.8% (95% CI 2.5 to 11.2) respectively.

### Students’ perception of the intervention

Most of the students in intervention schools (91.1%) said they liked the lessons a little or a lot (Table S3). Most (75.3%) said they found the lessons easy or very easy to understand and most (93.0%) reported that what they learned from the lessons was helpful or very helpful. These perceptions are corresponding with the results just after the intervention, when the respective proportions were 94.1, 78.5, and 94.0%.

## Discussion

In comparison to no intervention, the Informed Health Choices secondary school intervention improved students’ scores on the CHT test both immediately after the intervention term and 1 year later. However, there was some decay in what the students learned, and the size of the effects was smaller after 1 year. Retention for the primary outcome (a passing score on the CTH test) was 88%. Decay in learning is not unusual [34], and it may reflect a lack of practice. Other possible explanations were identified in the process evaluation conducted alongside the trial [24]. These included that some students were skeptical of some of the key concepts which challenged beliefs they had about treatments. Also, the key concepts were not included in the curriculum and not examined, so some students did not prioritize the *Be Smart About Your Health* lessons. These factors also may have contributed to some students not retaining what they had learned.

Teachers also benefitted from the intervention and there was less decay in the size of the effects after 1 year, 97% of teachers had a passing score and 91% had mastered the key concepts. This may reflect a greater intensity of the intervention for teachers (including the 2-day training workshop conducted before the intervention, preparing, and teaching the lessons), and perhaps possible that teachers were able to have more opportunities to apply the concepts in their daily lives and /or practice using what they learned [35]. Perhaps, is it also possible that teachers were able to link the concepts to previous training as part of their higher education?

Students in the intervention schools said the Informed Health Choice resources were helpful. They also were more likely than those in control schools to say they would find out the basis for a treatment claim and would find out if a treatment claim was based on research. In addition, they expressed more confidence in their ability to assess whether a treatment claim is based on research.

In the process evaluation linked to this trial, we found that the lessons were largely delivered as intended but required more time than planned and Swahili was frequently used by teachers although the lesson plans were in English [24]. None of the teachers changed schools during the school term when the lessons were taught, and we did not find any evidence of contamination.

A previous 1-year follow-up study of the Informed Health Choice primary school intervention in Uganda [36] found a sustained large effect of the intervention among children. Concurrent 1-year follow-up studies of the IHC secondary school intervention in Uganda and Rwanda had similar findings to this study. (In review) Potential reasons for better retention in the study are that the primary school intervention was more intense than the secondary school intervention. It included nine double period (80 min) lessons (12 h) compared to 10 40-min lessons (7 h) for the secondary school intervention. Additionally, the primary school intervention included textbooks and exercise books for the pupils, whereas the secondary school intervention did not include printable resources, but not printed resources or any other resources for students. Moreover, the secondary school intervention was implemented just after the pandemic, during a period when schools were focused on catching up with the usual regular curriculum after a long period of school closures.

There have been few other evaluations of secondary school interventions to improve people’s ability to think critically about the effects of health interventions, and those studies have not measured long-term retention, a year or more after the intervention [9, 10]. Strengths of this study include the large sample size, of sampled schools, and the length of the follow-up. Evaluation of the use of what was learned in daily life, and potential adverse effects, will be reported in separate studies [37].

A limitation of this study is the risk of bias due to a large loss to follow-up (27% of students and 16% of teachers). This was primarily due to teacher transfers mandated by the Ministry of Education and student school changes, both of which are common. To assess the impact of this loss, we conducted sensitivity analyses, and these indicate that our effect estimates are robust. As noted in the results, slightly more boys than girls were lost to follow-up in the intervention arm compared to the control arm. We investigated sex as a potential effect modifier in a meta-analysis of the three trials of the IHC secondary school intervention and found evidence that boys were more likely to achieve a passing score than girls (moderate credibility) [38]. Thus, losing more boys in the intervention arm may potentially have biased the results in favor of the control schools. However, the degree to which the results may have been biased and the direction of bias are uncertain. In the meta-analysis, two independent researchers assessed the risk of bias for the main outcomes in all three trials and the overall certainty of the evidence as moderate, due to missing outcome data.

Other limitations are similar to those described in the report of the results just after the intervention, [16] including that the outcome measure used was aligned with the intervention (“treatment-inherent”). Treatment-inherent outcome measures yield larger effect sizes than outcome measures that are not treatment-inherent [39, 40]. However, the CTH test drew on questions from a databank of items that independent researchers judged to have face validity, [41] we validated the outcome measure using Rasch analysis, [42] and an independent group of judges determined the standards for passing and mastery [26] . To mitigate the risk of teaching to the test, we did not show the CTH test to any of the teachers until it was administered after the 10 lessons were taught. We cannot rule out response bias, due to participants’ awareness of their group assignment, i.e., it is possible that participants in the intervention schools put more effort into completing the CTH test.

## Implications

We have shown reliably that it is possible to teach adolescents in secondary school in a low-income country to evaluate information about treatment effects. Results of a process evaluation conducted alongside this trial indicate that at least some students can and do use these skills in their daily lives (work in revision). However, a one-off intervention is unlikely to have large long-term effects on decision-making, health behaviors, or health. Highlighting follow-up training is likely necessary to reinforce these skills over time.

Potential strategies for sustaining the effects of the intervention, in addition to additional training or practice, include use of examples that do not challenge students’ or teachers’ beliefs about treatments before using examples that do, and more focus on the importance of being open minded. Importantly, incorporating the key concepts in the curriculum and examinations could help to ensure that the lessons are prioritized, thereby strengthening learning and retention.

## Supplementary Information

Supplementary Material 1.

## Data Availability

The de-identified dataset and data dictionary will be available on Zenodo with publication of this report, together with the protocol, informed consent forms, and outcome evaluation tool [13].

## References

[1] Oxman M, Larun L, Pérez Gaxiola G, Alsaid D, Qasim A, Rose CJ, et al. Quality of information in news media reports about the effects of health interventions: Systematic review and meta-analyses. F1000Research. 2022;10:433. 10.12688/f1000research.52894.1 PMC8756300 35083033

[2] Swire-Thompson B, Lazer D. Public health and online misinformation: Challenges and recommendations. Annu Rev Public Health. 2019;41:433–51. 31874069 10.1146/annurev-publhealth-040119-094127

[3] Dahlgren A, Furuseth-Olsen K, Rose CJ, Oxman AD. The Norwegian public’s ability to assess treatment claims: results of a cross-sectional study of critical health literacy [version 2; peer review: 1 approved, 2 approved with reservations]. F1000Research. 2021;9:179. 10.12688/f1000research.21902.2. 10.12688/f1000research.21902.2 PMC10995534 38585673

[4] Semakula D, Nsangi A, Oxman AD, Oxman M, Austvoll-Dahlgren A, Rosenbaum S, et al. Effects of the Informed Health Choices podcast on the ability of parents of primary school children in Uganda to assess claims about treatment effects: a randomised controlled trial. Lancet (London, England). 2017;390:389–98. 28539196 10.1016/S0140-6736(17)31225-4

[5] Roozenbeek J, Schneider CR, Dryhurst S, Kerr J, Freeman ALJ, Recchia G, van der Bles AM, van der LS. Susceptibility to misinformation about COVID-19 around the world. R Soc Open Sci. 2020;7201199. 10.1098/rsos.201199. PMC7657933 33204475

[6] Semakula D, Nsangi A, Oxman AD, Oxman M, Austvoll-Dahlgren A, Rosenbaum S, et al. Effects of the Informed Health Choices podcast on the ability of parents of primary school children in Uganda to assess the trustworthiness of claims about treatment effects: One-year follow up of a randomised trial. Trials. 2020;21:1–18. 32059694 10.1186/s13063-020-4093-x PMC7023790

[7] Brownlee S, Chalkidou K, Doust J, Elshaug AG, Glasziou P, Heath I, et al. Evidence for Overuse of Medical Services Around the World. Lancet. 2017;390:156. 28077234 10.1016/S0140-6736(16)32585-5 PMC5708862

[8] Glasziou P, Straus S, Brownlee S, Trevena L, Dans L, Guyatt G, et al. Evidence for underuse of effective medical services around the world. Lancet. 2017;390:169–77. 28077232 10.1016/S0140-6736(16)30946-1

[9] Cusack L, Del Mar CB, Chalmers I, Gibson E, Hoffmann TC. Educational interventions to improve people’s understanding of key concepts in assessing the effects of health interventions: a systematic review. Syst Rev. 2018;7:1–12. 29716639 10.1186/s13643-018-0719-4 PMC5930693

[10] Nordheim LV, Gundersen MW, Espehaug B, Guttersrud O, Flottorp S. Effects of school-based educational interventions for enhancing adolescents abilities in critical appraisal of health claims: a systematic review. PLoS ONE. 2016;11:e0161485. 27557129 10.1371/journal.pone.0161485 PMC4996462

[11] Agaba JJ, Chesire F, Mugisha M, Nandi P, Njue J, Nsangi A, et al. Prioritisation of Informed Health Choices (IHC) key concepts to be included in lower secondary school resources: a consensus study. PLoS ONE. 2023;18:e0267422. 37027357 10.1371/journal.pone.0267422 PMC10081733

[12] Chalmers I, Oxman AD, Austvoll-Dahlgren A, Ryan-Vig S, Pannell S, Sewankambo N, et al. Key Concepts for Informed Health Choices: a framework for helping people learn how to assess treatment claims and make informed choices. BMJ Evid-Based Med. 2018;23:29–33. 29367324 10.1136/ebmed-2017-110829

[13] Rosenbaum S, Moberg J, Chesire F, Mugisha M, Ssenyonga R, Ochieng MA, et al. Teaching critical thinking about health information and choices in secondary schools: human-centred design of digital resources. F1000Research. 2023;12:481. 39246586 10.12688/f1000research.132580.3 PMC11377934

[14] Rosenbaum S, Moberg J, Oxman M, Oxman AD, Chesire F, Mugisha M et al. Be smart about your health. 2023.

[15] Dahlgren A, Semakula D, Chesire F, Mugisha M, Nakyejwe E, Nsangi A, et al. Critical thinking about treatment effects in Eastern Africa: development and Rasch analysis of an assessment tool. F1000Research. 2023;12:887.

[16] Chesire F, Kaseje M, Ochieng M, Ngatia B, Mugisha M, Ssenyonga R, et al. Effects of the informed health choices secondary school intervention on the ability of students in Kenya to think critically about health choices: a cluster-randomized trial. J Evid Based Med. 2023;16:275–84. 37735827 10.1111/jebm.12556

[17] Chesire F, Kaseje M, Ochieng M, Ngatia B, Mugisha M, Ssenyonga R, et al. Effects of the informed health choices secondary school intervention on the ability of students in Kenya to think critically about health choices: a protocol for cluster-randomized trial. J Evid Based Med. 2023;16:275–84. 37735827 10.1111/jebm.12556

[18] Chesire F, Kaseje M, Ochieng M, Mugisha M, Ssenyonga R, Oxman M, et al. Effect of the Informed Health Choices digital secondary school resources on the ability of lower secondary students in Kenya to critically appraise health claims: protocol for a process evaluation. 2022.

[19] Mugisha M, Nyirazinyoye L, Simbi CMC, Chesire F, Senyonga R, Oxman M, et al. Effects of the Informed Health Choices secondary school intervention on the ability of students in Rwanda to think critically about health choices: A cluster-randomized trial. J Evid Based Med. 2023. 10.1111/JEBM.12551. 37735809 10.1111/jebm.12551

[20] Ssenyonga R, Oxman AD, Nakyejwe E, Chesire F, Mugisha M, Nsangi A, et al. Use of the informed health choices educational intervention to improve secondary students’ ability to think critically about health interventions in Uganda: A cluster-randomized trial. J Evid Based Med. 2023;16:285–93. 37725488 10.1111/jebm.12553

[21] Mugisha M, Oxman AD, Nyirazinyoye L, Uwitonze AM, Simbi CMC, Chesire F, Ssenyonga R, Oxman M, Nsangi A, Semakula D, Kaseje M, Sewankambo NK, Rosenbaum S, Lewin S. Process Evaluation of Teaching Critical Thinking About Health Using the Informed Health Choices Intervention in Rwanda: A Mixed Methods Study. Glob Health Sci Pract. 2024;12(6):e2300483. 10.9745/GHSP-D-23-00483. 10.9745/GHSP-D-23-00483 PMC11666086 39706678

[22] Ssenyonga R, Lewin S, Nakyejwe E, Chelagat F, Mugisha M, Oxman M, et al. Process evaluation of teaching critical thinking about health using the Informed Health Choices Intervention in Uganda: a mixed methods study. Glob Heal Sci Pract. 2024;12. 10.9745/GHSP-D-23-00484 PMC11666090 39706681

[23] Cambridge Clinical Trials Unit Box 401 Standard Operating Procedure CCTU/SOP58 Randomisation using Sealed Envelope.

[24] Chesire F, Oxman AD, Kaseje M, Gisore V, Mugisha M, Ssenyonga R, Oxman M, Nsangi A, Semakula D, Nyirazinyoye L, Sewankambo NK, Munthe-Kaas H, Holst C, Rosenbaum S, Lewin S. Process Evaluation of Teaching Critical Thinking About Health Using the Informed Health Choices Intervention in Kenya: A Mixed Methods Study. Glob Health Sci Pract. 2024;12(6):e2300485. 10.9745/GHSP-D-23-00485. 10.9745/GHSP-D-23-00485 PMC11666096 39706679

[25] Phillips AC, Lewis LK, McEvoy MP, Galipeau J, Glasziou P, Moher D, et al. Development and validation of the guideline for reporting evidence-based practice educational interventions and teaching (GREET). BMC Med Educ. 2016;16:1–10. 27599967 10.1186/s12909-016-0759-1 PMC5011880

[26] Nsangi A, Aranza D, Asimwe R, Munaabi-Babigumira SK, Nantongo J, Nordheim LV, et al. What should the standard be for passing and mastery on the Critical Thinking about Health Test? A consensus study BMJ Open. 2023;13:e066890. 36828652 10.1136/bmjopen-2022-066890 PMC9972413

[27] Nedelsky L. Absolute grading standards for objective tests. Educ Psychol Meas. 1954;14:3–19.

[28] Angoff WH. Educational Measurement. 2nd ed. American Council on Education/Macmillan Series in Higher Education. Washington, DC: American Council on Education; 1971. https://books.google.co.ke/books/about/Educational_Measurement.html?id=1HqdAAAAMAAJ&redir_esc=y.

[29] University of Aberdeen Health Services Research. Cluster sample size calculator user manual. Aberdeen Univ Aberdeen; 1999. https://www.abdn.ac.uk/media/site/ace/content-images/calculationmanual.pdf.

[30] Seaman SR, White IR. Review of inverse probability weighting for dealing with missing data. Stat Methods Med Res. 2013;22:278–95. 21220355 10.1177/0962280210395740

[31] Lee DS, Lee SD. Training, wages, and sample selection: estimating sharp bounds on treatment effects. Rev Econ Stud. 2009;76:1071–102.

[32] Zou H, Hastie T. Regularization and variable selection via the elastic net. J R Stat Soc: Series B (Statistical Methodology). 2005;67(2):301–20. 10.1111/j.1467-9868.2005.00503.x.

[33] Hedges LV. Effect Sizes in Cluster-Randomized Designs. 2016;32:341–70.

[34] Custers EJFM. Long-term retention of basic science knowledge: A review study. Adv Health Sci Educ. 2010;15:109–28. 10.1007/s10459-008-9101-y 18274876

[35] Arthur W, Bennett W, Stanush PL, McNelly TL. Factors that influence skill decay and retention: A quantitative review and analysis. Hum Perform. 1998;11:57–101.

[36] Nsangi A, Semakula D, Oxman AD, Austvoll-Dahlgren A, Oxman M, Rosenbaum S, Morelli A, Glenton C, Lewin S, Kaseje M, Chalmers I, Fretheim A, Ding Y, Sewankambo NK. Effects of the Informed Health Choices primary school intervention on the ability of children in Uganda to assess the reliability of claims about treatment effects, 1-year follow-up: a cluster-randomised trial. Trials. 2020;21(1):27. 10.1186/s13063-019-3960-9. 10.1186/s13063-019-3960-9 PMC6945419 31907013

[37] Oxman M, Chesire FC, Mugisha M, et al. Development of a framework of potential adverse effects of interventions to improve critical thinking about health choices: A mixed methods study [version 1; peer review: awaiting peer review]. F1000Research. 2024;13:1303. 10.12688/f1000research.158042.1. 10.12688/f1000research.158042.2 PMC12676117 41357787

[38] Chesire F, Mugisha M, Ssenyonga R, Rose CJ, Nsangi A, Kaseje M, et al. Effects of the informed health choices secondary school intervention after 1 year: a prospective meta-analysis using individual participant data. Trials. 2024;25:733. 39478569 10.1186/s13063-024-08577-w PMC11523815

[39] Abrami PC, Bernard RM, Borokhovski E, Waddington DI, Wade CA, Persson T. Strategies for Teaching Students to Think Critically. 2015;85:275–314. 10.3102/0034654314551063.

[40] Slavin R, Madden NA. Measures inherent to treatments in program effectiveness reviews. J Res Educ Eff. 2011;4:370–80.

[41] Austvoll-Dahlgren A, Semakula D, Nsangi A, Oxman AD, Chalmers I, Rosenbaum S, et al. Measuring ability to assess claims about treatment effects: the development of the ‘Claim Evaluation Tools.’ BMJ Open. 2017;7:e013184. 28515181 10.1136/bmjopen-2016-013184 PMC5777467

[42] Dahlgren A, Semakula D, Chesire F, et al. Critical thinking about treatment effects in Eastern Africa: development and Rasch analysis of an assessment tool [version 1; peer review: 1 approved with reservations]. F1000Research. 2023;12:887. 10.12688/f1000research.132052.1.

